# Enhanced Comprehensive Learning Particle Swarm Optimization with Dimensional Independent and Adaptive Parameters

**DOI:** 10.1155/2021/6628564

**Published:** 2021-02-05

**Authors:** Xiang Yu, Yu Qiao

**Affiliations:** ^1^Provincial Key Laboratory for Water Information Cooperative Sensing and Intelligent Processing, Nanchang Institute of Technology, Nanchang, Jiangxi 330099, China; ^2^School of Mathematics and Information Science, Shaanxi Normal University, Xi'an, Shaanxi 710119, China

## Abstract

Comprehensive learning particle swarm optimization (CLPSO) and enhanced CLPSO (ECLPSO) are two literature metaheuristics for global optimization. ECLPSO significantly improves the exploitation and convergence performance of CLPSO by perturbation-based exploitation and adaptive learning probabilities. However, ECLPSO still cannot locate the global optimum or find a near-optimum solution for a number of problems. In this paper, we study further bettering the exploration performance of ECLPSO. We propose to assign an independent inertia weight and an independent acceleration coefficient corresponding to each dimension of the search space, as well as an independent learning probability for each particle on each dimension. Like ECLPSO, a normative interval bounded by the minimum and maximum personal best positions is determined with respect to each dimension in each generation. The dimensional independent maximum velocities, inertia weights, acceleration coefficients, and learning probabilities are proposed to be adaptively updated based on the dimensional normative intervals in order to facilitate exploration, exploitation, and convergence, particularly exploration. Our proposed metaheuristic, called adaptive CLPSO (ACLPSO), is evaluated on various benchmark functions. Experimental results demonstrate that the dimensional independent and adaptive maximum velocities, inertia weights, acceleration coefficients, and learning probabilities help to significantly mend ECLPSO's exploration performance, and ACLPSO is able to derive the global optimum or a near-optimum solution on all the benchmark functions for all the runs with parameters appropriately set.

## 1. Introduction

Particle swarm optimization (PSO) [1, 2] is a powerful class of metaheuristics for global optimization. PSO simulates the social behavior of sharing individual knowledge when a flock of birds search for food. In PSO, flock and bird are, respectively, termed as swarm and particle, and each particle represents a candidate solution. Suppose the problem to be solved has *D* decision variables, each particle, denoted as *i*, “flies” in a *D*-dimensional search space and is accordingly associated with a *D*-dimensional velocity *V*_*i*_={*V*_*i*,1_, *V*_*i*,2_,…, *V*_*i*,*D*_}, a *D*-dimensional position *P*_*i*_={*P*_*i*,1_, *P*_*i*,2_,…, *P*_*i*,*D*_}, and a fitness *f* (*P*_*i*_) indicating the optimization performance of *P*_*i*_. The swarm of particles randomly initialize velocities and positions and search the global optimum iteratively, and the final solution found is the historical position that exhibits the best fitness value among all the particles. In each iteration (or generation), *i* updates *V*_*i*_ according to the present value of *V*_*i*_, the historical position giving *i*'s best fitness value so far (i.e., *i*'s personal best position), and the personal best positions of other particles.

Many different PSO variants have been proposed in the literature since the introduction of PSO in 1995 [3]. For the earliest proposed global PSO (GPSO) [3, 4], the global best position with the best fitness value among all the particles' personal best positions is used for particle velocity update. To be specific, in each generation, *i*'s velocity *V*_*i*_ and position *P*_*i*_ are adjusted on each dimension as follows:

(1) $$\begin{array}{c} V_{i,d}=wV_{i,d}+ar\left(B_{i,d}-P_{i,d}\right)+bs\left(G_{d}-P_{i,d}\right), \end{array}$$

(2) $$\begin{array}{c} P_{i,d}=P_{i,d}+V_{i,d}, \end{array}$$

where *d* (1 ≤ *d* ≤ *D*) is the dimension index; *w* is the inertia weight; *a* and *b* are the acceleration coefficients; *r* and *s* are two random numbers uniformly distributed in [0, 1]; *B*_*i*_={*B*_*i*,1_, *B*_*i*,2_,…, *B*_*i*,*D*_} is *i*'s personal best position; and *G*={*G*_1_, *G*_2_,…, *G*_*D*_} is the global best position. GPSO is liable to get stuck in a local optimum if the global best position is far from the global optimum. Local PSO (LPSO) [5] sets up a social topology with the shape of, e.g., ring, star, and pyramid. *i*'s neighborhood comprises *i* itself and the particles that are directly connected with *i* in the topology. Unlike GPSO, LPSO takes advantage of *i*'s local best position *L*_*i*_={*L*_*i*,1_, *L*_*i*,2_,…, *L*_*i*,*D*_} that gives the best fitness value among *i*'s neighborhood to guide the flight trajectory update of *i*, as can be seen from the following equation:

(3) $$\begin{array}{c} V_{i,d}=wV_{i,d}+ar\left(B_{i,d}-P_{i,d}\right)+bs\left(L_{i,d}-P_{i,d}\right). \end{array}$$

Compared with GPSO, LPSO reduces the chance of resulting in a local optimum. With respect to both GPSO and LPSO, *i*'s personal best position and the global/local best position are used for updating *V*_*i*_ on all the dimensions. However, the personal best position and the global/local best position actually do not always contribute to the velocity update on each dimension. Comprehensive learning PSO (CLPSO) [6] and orthogonal learning PSO (OLPSO) [7] encourage *i* to learn from different exemplars on different dimensions according to equation (4) when updating *V*_*i*_.

(4) $$\begin{array}{c} V_{i,d}=wV_{i,d}+ar\left(E_{i,d}-P_{i,d}\right), \end{array}$$

where *E*_*i*_={*E*_*i*,1_, *E*_*i*,2_,…, *E*_*i*,*D*_} is *i*'s exemplar position. In CLPSO, *i* is additionally associated with a fixed learning probability that controls *E*_*i,d*_ = *B*_*i*,*d*_ or *B*_*j*,*d*_ on each dimension *d*, where *j* is a particle randomly selected, and *j* ≠ *i*. OLPSO sets *E*_*i*,*d*_ as *i*'s personal best position or the global/local best position on each dimension *d* with the aid of orthogonal experimental design; OLPSO therefore has two versions: the global version OLPSO-G and the local version OLPSO-L. CLPSO and OLPSO redetermine *E*_*i*_ if *i*'s personal best fitness value *f* (*B*_*i*_) does not improve for a consecutive number of generations. CLPSO and OLPSO significantly outperform GPSO and LPSO in terms of preserving the particles' diversity and probing different regions of the search space to obtain a promising solution.

Metaheuristics including PSO need to address three important issues, namely, exploration, exploitation, and convergence. Exploration means searching diversely to locate a small region that possibly contains the global optimum, while exploitation refers to concentrating the search around the small region for solution refinement. Convergence is the gradual transition from initial exploration to ensuing exploitation. We studied enhancing the exploitation and convergence performance of CLPSO in [8], and our proposed PSO variant is called enhanced CLPSO (ECLPSO). ECLPSO calculates $\underline{B_{d}}$ and $\bar{B_{d}}$ which are, respectively, the lower bound and the upper bound of all the particles' personal best positions on each dimension *d* in each generation as follows:

(5) $$\begin{array}{c} {\underline{B}}_{d}=\min\left\{B_{1,d},B_{2,d},\ldots ,B_{N,d}\right\},\\ {\bar{B}}_{d}=\max\left\{B_{1,d},B_{2,d},\ldots ,B_{N,d}\right\}, \end{array}$$

where *N* is the number of particles. $\left[{\underline{B}}_{d},{\bar{B}}_{d}\right]$ is termed as the normative interval of dimension *d*. Let the search space on dimension *d* be $\left[{\underline{P}}_{d},{\bar{P}}_{d}\right]$ with $\underline{P_{d}}$ being the lower bound and ${\bar{P}}_{d}$ being the upper bound; ECLPSO deems that when $\left[{\underline{B}}_{d},{\bar{B}}_{d}\right]$ becomes indeed small (i.e., no greater than 1% of $\left[{\underline{P}}_{d},{\bar{P}}_{d}\right]$ and no greater than absolute value 2 simultaneously), the swarm of particles enter the exploitation phase on dimension *d* (i.e., the global optimum or a near-optimum solution has been identified to be likely around the normative interval on dimension *d*); otherwise, the particles are still in the exploration phase on dimension *d* (i.e., searching different regions on dimension *d*). ECLPSO adaptively updates the learning probability of each particle based on the ranking of all the particles' personal best fitness values and the number of the dimensions that have entered the exploitation phase. In addition, ECLPSO conducts perturbation on each dimension *d* that has entered the exploitation phase in order to find a high-quality solution around the normative interval on that dimension.

For a PSO variant, the velocity *V*_*i*,*d*_ of each particle *i* on each dimension *d* is usually clamped by a maximum velocity ${\bar{V}}_{d}$, i.e.,

(6) $$\begin{array}{c} V_{i,d}=\left\{\begin{array}{c} {\bar{V}}_{d},\text{if }V_{i,d}>{\bar{V}}_{d},\\ -{\bar{V}}_{d},\text{else if }V_{i,d}<-{\bar{V}}_{d},\\ V_{i,d},\text{otherwise}. \end{array}\right) \end{array}$$

If ${\bar{V}}_{d}$ was too large, the particles might miss some promising solutions on dimension *d*; on the contrary, too small ${\bar{V}}_{d}$ would slow down the search process on dimension *d*. ${\bar{V}}_{d}$ is fixed at 20% of the dimensional search space $\left[{\underline{P}}_{d},{\bar{P}}_{d}\right]$ by many literature PSO variants including GPSO, LPSO, CLPSO, OLPSO, and ECLPSO. The experimental results on various benchmark functions reported in [8] have demonstrated that though ECLPSO significantly improves the exploitation and convergence performance of CLPSO, it still cannot locate the global optimum or a near-optimum solution on a number of functions including Rosenbrock's function, rotated Schwefel's function, and rotated Rastrigin's function. The fight trajectory and search behavior of all the particles in ECLPSO are directly affected by each dimension *d*'s maximum velocity ${\bar{V}}_{d}$, the inertia weight *w*, the acceleration coefficient *a*, and each particle *i*'s learning probability *L*_*i*_. The experimental results reported in [8] have also indicated that the search process of the particles evolves differently on each dimension; hence, in this paper, we propose to assign an independent inertia weight and an independent acceleration coefficient corresponding to each dimension, as well as an independent learning probability for each particle on each dimension. The dimensional independent maximum velocities, inertia weights, acceleration coefficients, and learning probabilities are adaptively updated based on the dimensional normative intervals in order to facilitate exploration, exploitation, and convergence, particularly exploration. We call the variant with the dimensional independent and adaptive parameters as adaptive CLPSO (ACLPSO).

We note that existing PSO variants, e.g., [3–42], have rarely considered using dimensional independent parameters other than the dimensional independent maximum velocities, and we find only one work [26] as an exception. In [26], Taherkhani and Safabakhsh modified GPSO, CLPSO, and OLPSO with an independent inertia weight and an independent acceleration coefficient for each particle *i* on each dimension *d*; the inertia weight and the acceleration coefficient are adaptively adjusted according to the improvement status of *i*'s personal best fitness value and the distance between *i*'s dimensional position *P*_*i*,*d*_ and *i*'s dimensional personal best position *B*_*i*,*d*_ to achieve better exploration and faster convergence.

The rest of this paper is organized as follows.  Section 2 reviews the related work on PSO. The more detailed working principles of CLPSO and ECLPSO are elaborated in Section 3.  Section 4 presents our proposed dimensional independent and adaptive parameters and the space and time complexity analysis of ACLPSO. Performance evaluation of ACLPSO on a variety of benchmark functions is given in Section 5.  Section 6 concludes this paper.

## 2. Related Work

A lot of researchers worldwide have studied PSO. The status quo and research trend of PSO relevant research are to investigate multistrategy and adaptivity based on the 4 typical PSO variants, i.e., GPSO, LPSO, CLPSO, and OLPSO. Multistrategy refers to employing multiple strategies, while adaptivity stands for adaptively setting some parameters as well as appropriately invoking and switching the strategies. Multistrategy and adaptivity aim to realize goals such as exploration, exploitation, and convergence and help the particles efficiently find the global optimum or a near-optimum solution.

### 2.1. Related Work Based on GPSO/LPSO

Zhan [9] proposed adaptive GPSO; the variant identifies the swarm's evolution status based on the distribution of the distances among each particle and all the other particles; the inertia weight and acceleration coefficient are adaptively adjusted according to the swarm's evolution status for expediting convergence; the variant additionally takes advantage of Gaussian mutation to appropriately impose some momentum on the global best position to help escaping from a local optimum. Median-oriented GPSO was studied in [10]; the variant assigns an independent acceleration coefficient for each particle *i*; *i* is intentionally guided away from the swarm's median position that gives the median fitness value among all the particles' fitness values during the flight velocity update, and *i*'s associated acceleration coefficient is adaptively updated based on *i*'s fitness value, the swarm's worst fitness value, and the swarm's median fitness value so as to benefit jumping out of premature stagnancy in a local optimum and accelerating convergence. Chen et al. [11] introduced an aging mechanism with an aging leader and challengers for GPSO to address exploration; by evaluating the improvement status of the global best fitness value *f* (*G*), all the particles' personal best fitness values, and the leader's fitness value, the variant adaptively analyzes the leader's leading capability, adjusts the leader's life span, and generates a challenger through uniform mutation to possibly replace the leader when the leader's span becomes exhausted. GPSO augmented with multiple adaptive strategies was presented in [12]; nonuniform mutation and adaptive subgradient are alternatively applied to the global best position, respectively, contributing to escaping from a local optimum and conducting local search; the variant also performs Cauchy mutation on a randomly selected particle; as Cauchy mutation hinders convergence, the variant assigns an independent inertia weight and an independent acceleration coefficient for each particle and minimizes the sum of the distances between each particle and the global best position such that the inertia weights and acceleration coefficients are adaptively set and convergence is accordingly accelerated. In [13], LPSO with adaptive time-varying topology connectivity was investigated; for each particle *i*, the variant determines *i*'s historical contribution status to the global best position and the historical status of *i*'s topology connectivity getting stuck in a threshold value for every 5 consecutive generations and then adaptively updates *i*'s connectivity in the topology; the variant relies on neighborhood search to help the particles with their personal best fitness values ceasing improving in the present generation to jump out of stagnancy. Xia et al. [14] discussed GPSO with tabu detection and local search in a shrunk space; each dimension *d* is segmented into 7 regions of equivalent sizes; for every 5 consecutive generations, the variant calculates the excellence level of each region on dimension *d* based on the ranking of all the particles' personal fitness values and the distribution of all the particles' personal best positions in the regions; according to the excellence level of the region that the global best position belongs to, the variant appropriately randomly generates a possible replacement from some other region to assist escaping from a local optimum; when the global best position falls in a region on dimension *d* for 80 consecutive generations, the variant shrinks the dimensional search space to that specific region for the purpose of speeding up convergence; moreover, the variant conducts local search with the aid of differential evolution. Other recent works related to integrating GPSO/LPSO with multistrategy and/or adaptivity include [15–32].

### 2.2. Related Work Based on CLPSO/OLPSO

Liang and Suganthan [33] proposed adaptive CLPSO with history learning; for every 20 consecutive generations, the variant adaptively updates each particle's learning probability based on the best learning probability out of all the particles' learning probabilities (i.e., having resulted in the biggest improvement for the personal best fitness value) and Gaussian distribution. Memetic CLPSO was introduced in [34]; the variant employs chaotic local search to help each particle that cannot improve the personal best fitness value for 10 consecutive generations getting out of stagnancy and applies simulated annealing to the particle whose personal best fitness value continues improving for 3 consecutive generations and whose personal best position is actually the global best position for solution refinement. Zheng et al. [35] studied adaptively determining the inertia weight for CLPSO according to the relative ratio of the number of particles with improved personal fitness values in the present generation and adaptively setting the acceleration coefficient by considering the sum of the ratio of each particle's fitness change over the particle's position change in the present generation. Superior solution guided CLPSO was presented in [36]; for the variant, the set of superior solutions includes not only each particle's personal best position but also other historically experienced positions with excellent fitness values; each particle learns from the superior solutions for velocity update; the variant applies nonuniform mutation on each particle *i* to help escaping from a local optimum, and the mutation is activated only when *i*'s personal best fitness value ceases improving for 50 consecutive generations, and the average distance between *i*'s position in the present generation and *i*'s position in the previous 5 generation is less than a threshold value; the variant additionally takes advantage of some local search techniques (e.g., quasi-Newton, pattern search, and simplex search) to refine the global best position after 80% of the search progress. Qin et al. [37] investigated 4 auxiliary strategies for OLPSO to generate an appropriate exemplar position, respectively, for the purpose of preserving diversity, jumping out of premature stagnancy, accelerating convergence, and local search; the variant mutates the global best position to further strengthen exploration. Other recent works including [38–42] are also related to multistrategy and/or adaptivity research based on CLPSO/OLPSO.

## 3. Background

### 3.1. Comprehensive Learning Particle Swarm Optimization

In equation (4), the inertia weight *w* linearly decreases in each generation, and the acceleration coefficient *a* is a constant value equivalent to 1.5. Let *k*_max_ be the predefined maximum number of generations; *w* is updated in each generation *k* according to the following equation:

(7) $$\begin{array}{c} w=w_{\max}-\frac{k}{k_{\max}}\left(w_{\max}-w_{\min}\right), \end{array}$$

where *w*_max_=0.9 and *w*_min_=0.4 are, respectively, the maximum and minimum inertia weights.

Equation (8) is the empirical expression for setting each particle *i*'s learning probability *L*_*i*_. All the particles are associated with different learning probabilities.

(8) $$\begin{array}{c} L_{i}=L_{\min}+\left(L_{\max}-L_{\min}\right)\frac{\exp\left(10\left(i-1\right)|/|N-1\right)-1}{\exp\left(10\right)-1}, \end{array}$$

where *L*_max_ = 0.5 is the maximum learning probability and *L*_min_ = 0.05 is the minimum learning probability.

For each particle *i* on each dimension *d*, a random number uniformly distributed in [0, 1] is generated; if the number is no less than *L*_*i*_, the dimensional exemplar *E*_*i*,*d*_ = *B*_*i*,*d*_; otherwise, *E*_*i*,*d*_ = *B*_*j*,*d*_ with *j* ≠ *i*. To determine *j*, two different particles excluding *i* are randomly selected, and *j* is the winner with a better fitness value out of the two particles. If *E*_*i*,*d*_ is the same as *B*_*i*,*d*_ on all the dimensions, CLPSO randomly chooses one dimension to learn from some other particle's personal best position. CLPSO redetermines *i*'s exemplar position *E*_*i*_ if *i*'s personal best fitness value ceases improving for 7 consecutive generations.

CLPSO calculates the fitness value of *i* only if *i* is feasible (i.e., within the dimensional search space $\left[{\underline{P}}_{d},{\bar{P}}_{d}\right]$ on each dimension *d*). If *i* is infeasible, as all the dimensional exemplars are feasible, *i* will eventually be drawn back to the search space.

### 3.2. Enhanced Comprehensive Learning Particle Swarm Optimization

ECLPSO introduces two enhancements, namely, perturbation-based exploitation (PbE) and adaptive learning probabilities (ALPs), to improve the exploitation and convergence performance of CLPSO.

In each generation, regarding each dimension *d*, if the dimensional normative interval $\left[{\underline{B}}_{d},{\bar{B}}_{d}\right]$ is indeed small, the PbE enhancement updates the dimensional position *V*_*i*,*d*_ for each particle *i* according to equation (9) instead of equation (4):

(9) $$\begin{array}{c} V_{i,d}=w_{PbE}V_{i,d}+a_{PbE}r\left(E_{i,d}+c\left(\frac{{\underline{B}}_{d}+{\bar{B}}_{d}}{2}-E_{i,d}\right)-P_{i,d}\right), \end{array}$$

where *w*_PbE_=0.5 is the inertia weight used exclusively for the PbE enhancement; *a*_PbE_ = 1.5 is the acceleration coefficient used exclusively for the PbE enhancement; and *c* is the perturbation coefficient. *c* is randomly generated from a Gaussian distribution with mean 1 and standard deviation 0.65, and *c* is clamped to 10 times of the standard deviation on both sides of the mean. Each particle *i* is pulled towards *E*_*i*,*d*_ plus a perturbation term $c\left(\left({\underline{B}}_{d}+{\bar{B}}_{d}\right)/2-E_{i,d}\right)$ on dimension *d*. The PbE enhancement contributes to sufficient exploitation around the indeed small dimensional normative interval. Note that *V*_*i*,*d*_ updated by equation (9) is not limited by the dimensional maximum velocity ${\bar{V}}_{d}$.

The minimum learning probability *L*_min_ is fixed at 0.05. As expressed in equation (10), the maximum learning probability *L*_max_ logarithmically increases in each generation *k*:

(10) $$\begin{array}{c} L_{\max}=L_{\min}+h+q\;\log_{\left(D+1\right)}\left(M_{k}+1\right), \end{array}$$

where *M*_*k*_ is the number of exploitation valid dimensions (i.e., the number of the dimensions whose normative intervals have ever become indeed small) before or just in generation *k*; *h* = 0.25 is the difference coefficient; and *q* = 0.45 is the rate coefficient. *L*_max_ is small (i.e., 0.3) when *M*_*k*_ = 0 and benefits initial exploration. *L*_max_ increases rapidly with the particles' exploitation progress to facilitate convergence. The ALP enhancement adaptively determines all the particles' learning probabilities based on the ranking of the particles' personal best fitness values, i.e.,

(11) $$\begin{array}{c} L_{i}=L_{\min}+\left(L_{\max}-L_{\min}\right)\frac{\exp\left(10\left(T_{i}-1\right)/N-1\right)-1}{\exp\left(10\right)-1}, \end{array}$$

where *T*_*i*_ is *i*'s rank. If *i* gives the best personal best fitness value, then *T*_*i*_ = 1. A low-ranked particle is often better on more dimensions with respect to the personal best position than a high-ranked particle.

## 4. Adaptive Comprehensive Learning Particle Swarm Optimization

### 4.1. Dimensional Independent and Adaptive Maximum Velocities

**Figure 1:**
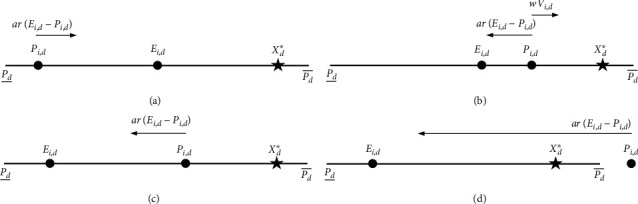
Illustration of why it is difficult for particle *i* to reach the dimensional global optimum *X*_*D*_^*∗*^ on dimension *d*. (a) *P*_*i*,*d*_ and *X*_*D*_^*∗*^ are close to different bounds of $\left[{\underline{P}}_{d},{\bar{P}}_{d}\right]$, and *E*_*i*,*d*_ is located in between *P*_*i*,*d*_ and *X*_*D*_^*∗*^. (b) *i* flies past *E*_*i*,*d*_. (c) *X*_*D*_^*∗*^ is around ${\bar{P}}_{d}$, *P*_*i*,*d*_ is not that far from *X*_*D*_^*∗*^, and *E*_*i*,*d*_ is close to $\underline{P_{d}}$. (d) *X*_*D*_^*∗*^ is around ${\bar{P}}_{d}$, *P*_*i*,*d*_ trespasses ${\bar{P}}_{d}$, and *E*_*i*,*d*_ is close to ${\underline{P}}_{d}$.

Suppose the optimization problem to be solved is *f* (*X*) with *X* being the *D*-dimensional decision vector, and the global optimum is *X*^*∗*^=(*X*_1_^*∗*^, *X*_2_^*∗*^,…, *X*_*D*_^*∗*^). CLPSO and ECLPSO fail to observe and address the fact that, on a dimension *d*, if the dimensional global optimum *X*_*D*_^*∗*^ is located near either bound of the dimensional search space $\left[{\underline{P}}_{d},{\bar{P}}_{d}\right]$ and all the particles' dimensional personal best positions are scattered (i.e., the dimensional normative interval $\left[{\underline{B}}_{d},{\bar{B}}_{d}\right]$ is large), then it would be difficult for the swarm of particles to locate *X*_*D*_^*∗*^; this is because the dimensional maximum velocity ${\bar{V}}_{d}$ updated by equation (4) is restricted to be 20% of $\left[{\underline{P}}_{d},{\bar{P}}_{d}\right]$.  Figure 1 illustrates this phenomenon. In Figure 1(a), a particle *i*'s dimensional position *P*_*i*,*d*_ and *X*_*D*_^*∗*^ are close to different bounds of $\left[{\underline{P}}_{d},{\bar{P}}_{d}\right]$, and *i*'s dimensional exemplar position *E*_*i*,*d*_ is located in between *P*_*i*,*d*_ and *X*_*D*_^*∗*^; the distance *E*_*i*,*d*_–*P*_*i*,*d*_ and the distance *X*_*D*_^*∗*^ − *E*_*i*,*d*_ both are equal to 40% of ${\bar{P}}_{d}-{\underline{P}}_{d}$; and *i* needs at least 2 generations to reach around *E*_*i*,*d*_. As can be seen from Figure 1(b), when *i* flies past *E*_*i*,*d*_, *i*'s dimensional velocity update is influenced by two forces, i.e., the inertia force *wV*_*i*,*d*_ and the exemplar force *ar* (*E*_*i*,*d*_–*P*_*i*,*d*_); the more *i* being away from *E*_*i*,*d*_, the more the exemplar force is to pull it back to *E*_*i*,*d*_. In Figure 1(c), *X*_*D*_^*∗*^ is around ${\bar{P}}_{d}$, and *P*_*i*,*d*_ is not that far from *X*_*D*_^*∗*^; however, *E*_*i*,*d*_ is close to $\underline{P_{d}}$; *E*_*i*,*d*_ guides *i* to fly away from *X*_*D*_^*∗*^. As a result, the chance for a particle to reach close to the dimensional global optimum is small. Furthermore, in case the dimensional global optimum is located near the dimensional search space bound and the dimensional normative interval is large on a significant number of dimensions, CLPSO and ECLPSO would fail to find the global optimum or a near-optimum solution, e.g., on Rosenbrock's function, rotated Schwefel's function, and rotated Rastrigin's function, for all the runs as reported in [8]. Therefore, ${\bar{V}}_{d}$ should not be fixed at 20% of $\left[{\underline{P}}_{d},{\bar{P}}_{d}\right]$. We propose to adaptively adjust ${\bar{V}}_{d}$ in each generation according to the following equation:

(12) $$\begin{array}{c} {\bar{V}}_{d}=s\left({\bar{B}}_{d}-{\underline{B}}_{d}\right), \end{array}$$

where *s* is the scaling coefficient and is a positive value. ${\bar{V}}_{d}$ is positively related with the dimensional normative interval's size ${\bar{B}}_{d}-{\underline{B}}_{d}$. When $\left[{\underline{B}}_{d},{\bar{B}}_{d}\right]$ is large, ${\bar{V}}_{d}$ is large and contributes to timely flight for getting close to *X*_*D*_^*∗*^; on the contrary, ${\bar{V}}_{d}$ is small when $\left[{\underline{B}}_{d},{\bar{B}}_{d}\right]$ becomes small in order to benefit fine-grained search.

Allowing each particle *i*'s position *P*_*i*,*d*_ on each dimension *d* to be infeasible also inhibits the particles to move close to *X*_*D*_^*∗*^.  Figure 1(d) shows an example; *X*_*D*_^*∗*^ is near ${\bar{P}}_{d}$, *P*_*i*,*d*_ trespasses ${\bar{P}}_{d}$ and is infeasible, and *E*_*i*,*d*_ is around ${\underline{P}}_{d}$; because of the force imposed by *E*_*i*,*d*_, *P*_*i*,*d*_ is pulled to a feasible dimensional position far from *X*_*D*_^*∗*^. Accordingly, an infeasible dimensional position is proposed to be repaired immediately by reinitialization between the previous feasible dimensional position and the trespassed dimensional search space bound [43, 44].

### 4.2. Dimensional Independent and Adaptive Inertia Weights and Acceleration Coefficients

For CLPSO and ECLPSO, the inertia weight *w* used in equation (4) is initially large to result in a large inertia force and is helpful for exploration, and *w* linearly decreases in each generation to gradually decrease the inertia force for the purpose of facilitating convergence and solution refinement. As *w* is dynamically updated according to the generation counter *k* in equation (7), *w* might obstruct exploration if the swarm of particles had not found the global optimum or a near-optimum solution even when *k* is large, and *w* might also impede convergence if a promising solution had already been located, and the particles can thus start solution refinement even when *k* is small. In addition, the same *w* is used with respect to all the dimensions. The search processes of the particles often evolve differently on different dimensions, i.e., taking different number of generations for the exploration phase. We thus propose to assign an independent weight *w*_*d*_ for each dimension *d* to replace *w* in equation (4) and adaptively set *w*_*d*_ when the dimensional normative interval $\left[{\underline{B}}_{d},{\bar{B}}_{d}\right]$is greater than 1% of the dimensional search space $\left[{\underline{P}}_{d},{\bar{P}}_{d}\right]$ or greater than 2 as follows:

(13) $$\begin{array}{c} w_{d}=u\frac{{\bar{B}}_{d}-\underline{B_{d}}}{{\bar{P}}_{d}-\underline{P_{d}}}+\left(1-u\right)\left(w_{\max}-\frac{k}{k_{\max}}\left(w_{\max}-w_{\min}\right)\right), \end{array}$$

(14) $$\begin{array}{c} w_{d}=\left\{\begin{array}{c} w_{\max},\text{if }w_{d}>w_{\max},\\ w_{\min},\text{else if }w_{d}<w_{\min},\\ w_{d},\text{otherwise,} \end{array}\right) \end{array}$$

where *u* is the tradeoff coefficient and is a positive number. *u* adjusts the tradeoff between the term $\left({\bar{B}}_{d}-{\underline{B}}_{d}\right)/\left({\bar{P}}_{d}-{\underline{P}}_{d}\right)$ and the term *w*_max_ − (*k*/*k*_max_)(*w*_max_ − *w*_min_). The empirical value chosen for *u* is 0.3. The incorporation of the term $\left({\bar{B}}_{d}-{\underline{B}}_{d}\right)/\left({\bar{P}}_{d}-{\underline{P}}_{d}\right)$ aims to improve the particles' exploration and convergence capabilities. If the dimensional normative interval $\left[{\underline{B}}_{d},{\bar{B}}_{d}\right]$ is large, the particles are still exploring different regions of the dimensional search space, and accordingly, *w*_*d*_ needs to be large; when $\left[{\underline{B}}_{d},{\bar{B}}_{d}\right]$ becomes small, *w*_*d*_ also grows small to facilitate convergence.

We further propose to assign an independent acceleration coefficient *a*_*d*_ for each dimension *d* to replace *a* in equation (4). *w*_*d*_ and *a*_*d*_ must satisfy the following so-called stability condition [26, 45, 46]:

(15) $$\begin{array}{c} 1-w_{d}\geq 0\text{ and }w_{d}+1-ra_{d}\geq 0. \end{array}$$

Hence, *a*_*d*_ is simply adaptively adjusted according to the following equation:

(16) $$\begin{array}{c} a_{d}=w_{d}+1. \end{array}$$

### 4.3. Dimensional Independent and Adaptive Learning Probabilities

Regarding each particle *i* in CLPSO and ECLPSO, a large value for *i*'s learning probability *L*_*i*_ enables *i* to learn more from its own personal best position for velocity update and hence is beneficial for solution refinement, while a small value for *L*_*i*_ will let *i* to learn more from other particles' personal best positions and accordingly encourages *i* to search diversely. *L*_*i*_ is adaptively updated based on *i*'s fitness rank *T*_*i*_ and the number of exploitation valid dimensions *M*_*k*_ in each generation *k*. A serious issue occurs if *M*_*k*_ is 0 or a small value in all the generations, e.g., as reported on Rosenbrock's function, rotated Schwefel's function, and rotated Rastrigin's function in [8]; small *M*_*k*_ leads to small learning probabilities for the swarm of particles and fails to realize convergence. We propose to assign an independent learning probability *L*_*i*,*d*_ for each particle *i* on each dimension *d* and adaptively set *L*_*i*,*d*_ in each generation *k* as follows:

(17) $$\begin{array}{c} L_{i,d}=v\;\log_{k_{\max}}\;k+\frac{{\bar{B}}_{d}-{\underline{B}}_{d}}{{\bar{P}}_{d}-{\underline{P}}_{d}}\frac{\exp\left(D\left(T_{i}-1\right)/N-1\right)-1}{\exp\left(D\right)-1}, \end{array}$$

(18) $$\begin{array}{c} L_{i,d}=\left\{\begin{array}{c} L_{\max},\text{if }L_{i,d}>L_{\max},\\ L_{\min},\text{ else if }L_{i,d}<L_{\min},\\ L_{i,d},\text{ otherwise,} \end{array}\right) \end{array}$$

where *L*_min_ = 0.05; *L*_max_ = 0.75; and *v* is the learning probability-based coefficient and is a positive number no greater than 1. The term log_*k*_max__  *k* grows logarithmically with the generator counter *k* in order to facilitate convergence. The term $\left({\bar{B}}_{d}-{\underline{B}}_{d}\right)/\left({\bar{P}}_{d}-{\underline{P}}_{d}\right)$, being positively related with ${\bar{B}}_{d}-{\underline{B}}_{d}$, also benefits convergence when the dimensional normative interval $\left[{\underline{B}}_{d},{\bar{B}}_{d}\right]$ is large.

### 4.4. Workflow and Complexity Analysis

ACLPSO is our proposed PSO variant based on ECLPSO with dimensional independent and adaptive maximum velocities, inertia weights, acceleration coefficients, and learning probabilities. The detailed step-by-step workflow of ACLPSO is as follows:

  Step 1: for each particle *i* and each dimension *d*, randomly initialize *i*'s dimensional velocity *V*_*i*,*d*_ and dimensional position *P*_*i*,*d*_ based on the dimensional search space $\left[{\underline{P}}_{d},{\bar{P}}_{d}\right]$, calculate *i*'s fitness value *f* (*P*_*i*_), and set *i*'s personal best fitness value *f* (*B*_*i*_) = *f* (*P*_*i*_), *i*'s dimensional personal best position *B*_*i*,*d*_ = *P*_*i*,*d*_, *i*'s cessation counter *W*_*i*_ = 0, the generation counter *k* = 1, the maximum number of generations *k*_max_, and all the other parameters
  Step 2: if *k* ≤ *k*_max_, go to Step 3; otherwise, go to Step 7
  Step 3: for each dimension *d*, determine the dimensional normative interval $\left[{\underline{B}}_{d},{\bar{B}}_{d}\right]$, and update the dimensional maximum velocity ${\bar{V}}_{d}$ according to equation (12)
  Step 4: for each particle *i*, calculate *i*'s fitness rank *T*_*i*_; and if *W*_*i*_% (*g* + 1) = 0, reset *W*_*i*_ = 1 and reassign *i*'s dimensional exemplar *E*_*i*,*d*_ on each dimension *d*
  Step 5: for each particle *i* and each dimension *d*, adjust *i*'s dimensional learning probability *L*_*i*,*d*_ according to equations (17) and (18); if $\left[{\underline{B}}_{d},{\bar{B}}_{d}\right]$ is indeed small, update *V*_*i*,*d*_ according to equation (9); otherwise, update the dimensional inertia weight *w*_*d*_ according to equations (13) and (14), the dimensional acceleration coefficient *a*_*d*_ according to equation (16), and *V*_*i*,*d*_ according to equations (4) and (6); update *P*_*i*,*d*_ according to equation (2), and repair *P*_*i*,*d*_ if *P*_*i*,*d*_ trespasses $\left[{\underline{P}}_{d},{\bar{P}}_{d}\right]$
  Step 6: for each particle *i*, calculate *f* (*P*_*i*_); if *f* (*P*_*i*_) ≥ *f* (*B*_*i*_), update *W*_*i*_ = *W*_*i*_ + 1; otherwise, set *f* (*B*_*i*_) = *f* (*P*_*i*_) and *B*_*i*,*d*_ = *P*_*i*,*d*_ on each dimension *d*
  Step 7: output the global best position with the best fitness value among all the particles' personal best positions

As analyzed in [8], the time and space complexities of ECLPSO are, respectively, *O* (*ND*) bytes and *O* (*k*_max_ (*N*log*N* + *ND*)) basic operations plus *O* (*k*_max_*N*) function evaluations (FEs). Concerning ACLPSO, storing the dimensional independent inertia weights and acceleration weights requires *O* (*D*) bytes, and storing the dimensional independent learning probabilities needs *O* (*ND*) bytes. Adaptively updating the dimensional independent maximum velocities, inertia weights, and acceleration coefficients calls for *O* (*k*_max_*D*) basic operations, and adaptively adjusting the dimensional independent learning probabilities demands for *O* (*k*_max_*ND*) basic operations. Therefore, the time and space complexities of ACLPSO are the same as those of ECLPSO.

## 5. Experimental Studies

### 5.1. Experimental Settings

The experimental hardware platform is a Microsoft Surface Pro laptop computer with an Intel Core i5-7300U central processor at the frequency of 2.6 GHz, 8 GB internal memory, and 256 GB solid-state disk external memory. The operating system is 64 bit Windows 10.

16 commonly studied 30-dimensional functions [6–8, 24, 47] are used in this paper for benchmarking ACLPSO and other PSO variants. The name, the expression, the global optimum, the function value of the global optimum, the search space, and the initialization space of each function are listed in Table 1. The functions are classified into 5 categories, namely, unimodal, multimodal, shifted, rotated, and shifted rotated. Rosenbrock's function *f*_3_ is unimodal in a 2-dimensional or 3-dimensional search space, but is multimodal in higher-dimensional cases [48]; it features a narrow valley from perceived local optima to the global optimum. With the incorporation of the cosine term cos(2*πX*_*d*_), there are a large number of regularly distributed local optima for Rastrigin's function *f*_5_. Ackley's function *f*_7_ has a deep global optimum and many minor local optima. Griewank's function *f*_8_ contains a cosine multiplication term $\prod_{d=1}^{D}\cos\left(X_{d}|/|\sqrt{d}\right)$ that causes linkages among the decision variables; *f*_8_ is similar to *f*_5_ in terms of many regularly distributed local optima. Schwefel's function *f*_9_ has a global optimum that is distant from the local optima. With respect to the unimodal and multimodal functions *f*_1_ to *f*_9_, the dimensional values of the global optimum are the same on all the dimensions. A shifted function shifts the global optimum *X*^*∗*^ to a vector *Z* that can be different on each dimension. A rotated function multiplies the original decision vector *X* by an orthogonal matrix *O* to get a rotated decision vector *Y* = *XO*; because of the rotation, if one dimension of *X* changes, all the dimensions of *Y* get affected. A shifted rotated function is both shifted and rotated. The shifted global optima of the shifted functions *f*_10_ to *f*_12_ and the shifted rotated function *f*_16_ can be found in [47]. The orthogonal matrices of the rotated functions *f*_13_ to *f*_15_ and the shifted rotated function *f*_16_ are generated by Salomon's method [49]. The initialization spaces of the functions *f*_1_, *f*_2_, *f*_4_, *f*_5_, *f*_6_, *f*_7_, *f*_8_, *f*_13_, and *f*_15_ are intentionally set to be asymmetric.

We conduct experiments to investigate the following 3 issues: (1) what are the key parameters of ACLPSO and how do the key parameters impact the performance of ACLPSO? (2) How do the dimensional independent and adaptive maximum velocities, inertia weights, acceleration coefficients, and learning probabilities improve the performance of ACLPSO? (3) How is the performance of ACLPSO as compared with other PSO variants? We consider 3 variants of ACLPSO, i.e., ACLPSO-1, ACLPSO-2, and ACLPSO-3. ACLPSO-1, ACLPSO-2, and ACLPSO-3 are the same as ACLPSO, except that ACLPSO-1 does not repair the dimensional position *P*_*i*,*d*_ for each particle *i* on each dimension *d* if *P*_*i*,*d*_ trespasses the dimensional search space $\left[{\underline{P}}_{d},{\bar{P}}_{d}\right]$, ACLPSO-2 does not adopt the dimensional independent and adaptive inertia weights and acceleration coefficients, and ACLPSO-3 does not take advantage of the dimensional independent and adaptive learning probabilities. Besides ACLPSO-1, ACLPSO-2, and ACLPSO-3, ACLPSO is further compared with CLPSO [6], ECLPSO [8], OLPSO-L [7], adaptive GPSO (AGPSO) [9], feedback learning GPSO with quadratic inertia weight (FLGPSO-QIW) [15], and GPSO with an aging leader and challengers (ALC-GPSO) [11]. For ACLPSO, ACLPSO-1, ACLPSO-2, ACLPSO-3, CLPSO, and ECLPSO, they are all implemented by Java, the number of particles *N* is set as 40, and 25 runs are executed on each function. The parameters of CLPSO, ECLPSO, OLPSO-L, AGPSO, FLGPSO-QIW, and ALC-GPSO take the recommended values that were empirically determined based on extensive experiments on various benchmark functions in [6–9, 11, 15]. Note that the value of *N* could be different for different PSO variants. *N* is fixed at 40 for CLPSO, ECLPSO, and OLPSO-L in [6–8], while it is equal to 20 for AGPSO, FLGPSO-QIW, and ALC-GPSO in [9, 11, 15]. As we do not have the source codes of OLPSO-L, AGPSO, FLGPSO-QIW, and ALC-GPSO, we directly copy the results of these 4 variants from [7, 24] for performance comparison. Concerning all the PSO variants compared, each run consumes 200,000 FEs.

### 5.2. Experimental Results and Discussion

**Figure 2:**
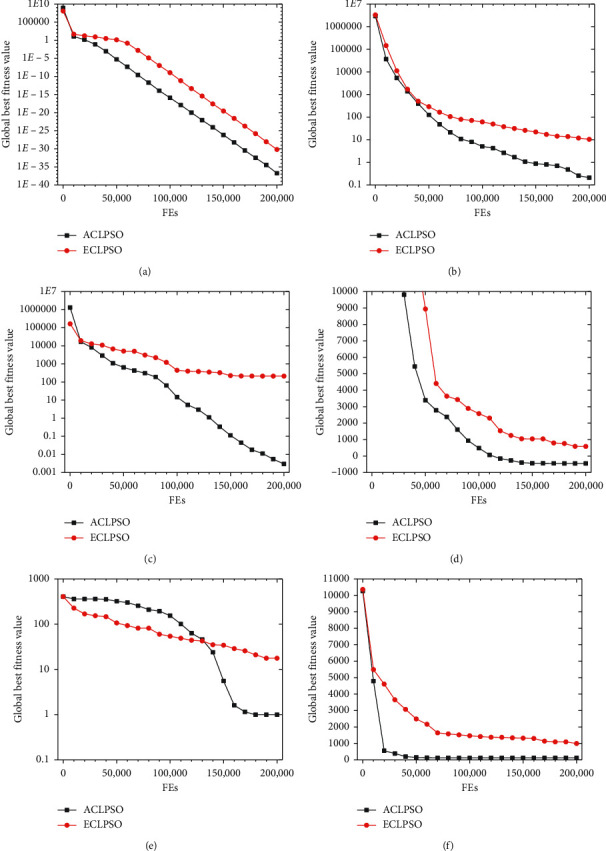
The changes of the global best fitness value during the search process of ACLPSO with the best (*s*, *v*) combination and in the best run. (a) *f*_2_. (b) *f*_3_. (c) *f*_4_. (d) *f*_12_. (e) *f*_13_. (f) *f*_14_.

The mean and standard deviation (SD) global best fitness value results of ACLPSO with different combinations for the values of the normative interval scaling coefficient *s* and the learning probability-based coefficient *v* on all the benchmark functions are listed in Table 2. Four different combinations are considered, i.e., I (*s* = 1.1, *v*=0.05), II (*s* = 1.1, *v*=0.3), III (*s* = 0.1, *v*=0.05), and IV (*s* = 0.1, *v*=0.3), and the best combination on each function is marked in bold.  Table 3 gives the mean and SD final number of exploitation valid dimensions' (i.e., *M*_*k*_max__) results of ACLPSO with 4 different (*s*, *v*) combinations on all the functions. The mean and SD global best fitness value results of ACLPSO with the best (*s*, *v*) combination, ACLPSO-1, ACLPSO-2, ACLPSO-3, CLPSO, ECLPSO, OLPSO-L, AGPSO, FLGPSO-QIW, and ALC-GPSO on all the functions are compared in Table 4. A two-tailed *t*-test with degree of freedom 48 and significance level 0.05 is performed between the global best fitness value results of ACLPSO with the best (*s*, *v*) combination and those of ECLPSO on each function, and the *t*-test results on all the functions are listed in Table 5.  Table 6 gives the mean and SD execution time results of ACLPSO with the best (*s*, *v*) combination, ECLPSO, and CLPSO on all the functions.  Table 7 lists the mean and SD global best fitness value results of ACLPSO with other parameter settings on *f*_3_, *f*_4_, *f*_12_, and *f*_14_. The mean and SD global best fitness value and execution time results of CLPSO with other parameter settings on *f*_1_ and *f*_2_ are given in Table 8.  Figure 2 illustrates the changes of the global best fitness value during the search process of ACLPSO with the best (*s*, *v*) combination and in the best run on *f*_2_, *f*_3_, *f*_4_, *f*_12_, *f*_13_, and *f*_14_.

As can be seen from Table 2, the best (*s*, *v*) combinations are, respectively, IV, IV, III, IV, I, II, II, II, I, I, II, IV, II, II, II, and IV on the 16 functions. ACLPSO with the best (*s*, *v*) combination is able to find the global optimum or a near-optimum solution on each function for all the 25 runs. ACLPSO is likely to get trapped in an unsatisfactory local optimum on *f*_3_ with combinations II and IV, on *f*_4_ with combinations I, II, and III, on *f*_5_ with combinations II and IV, on *f*_9_ with combinations II, III, and IV, on *f*_10_ with combinations II and IV, on *f*_12_ with combinations I and II, on *f*_13_ with combinations I, III, and IV, and on *f*_14_ with combinations III and IV. The accuracy of the mean global best fitness value with the best (*s*, *v*) combination is noticeably excellent on *f*_1_, *f*_2_, *f*_5_, *f*_6_, *f*_7_, *f*_8_, *f*_11_, *f*_15_, and *f*_16_. The observations indicate that *s* and *v* are the key parameters for ACLPSO, and the performance of ACLPSO is sensitive to the values of *s* and *v*. The normative interval scaling coefficient *s* determines the search granularity; large *s* encourages the particles to search in a large granularity so as to escape from an unsatisfactory local optimum and locate the global optimum or a near-optimum solution, whereas small *s* will let the particles to search in a small granularity so as not to miss the deep global optimum or a deep near-optimum solution during the search process. The learning probability-based coefficient *v* controls for a particle the number of dimensions learning from the particle's own personal best position; large and small values for *v*, respectively, contribute to the exchange of valuable information among the particles and preserving valuable information embodied in each particle; large *v* also benefits convergence and exploitation but might lead to premature stagnancy and hinders exploration in case some valuable information about the global optimum or a near-optimum solution was not preserved. We can see from Table 3 that the mean number of exploitation valid dimensions' results of ACLPSO with *v* being set as 0.3 is 30 or close to 30 on all the functions except *f*_6_; in contrast, the mean number of exploitation valid dimensions' results of ACLPSO is less than 11 or even 0 with *v* being fixed at 0.05 on *f*_3_, *f*_4_, *f*_6_, *f*_8_, *f*_10_, *f*_11_, *f*_12_, *f*_13_, *f*_14_, and *f*_15_. The functions have different landscapes; thus, different (*s*, *v*) combinations achieve the best performance on different functions.

In Table 4, ACLPSO-1, ACLPSO-2, and ACLPSO-3 take the same values for *s* and/or *v* as the best (*s*, *v*) combination of ACLPSO on each function. The mean global best fitness value results of ACLPSO-1 are worse than those of ACLPSO on *f*_1_, *f*_2_, *f*_3_, *f*_10_, *f*_13_, *f*_14_, *f*_15_, and *f*_16_. ACLPSO-2 performs inferior to ACLPSO in terms of the mean global best fitness value results on *f*_1_, *f*_2_, *f*_3_, *f*_4_, *f*_5_, *f*_10_, *f*_11_, *f*_12_, *f*_13_, *f*_14_, *f*_15_, and *f*_16_. The mean global best fitness value results of ACLPSO-3 are also worse than those of ACLPSO on all the functions except *f*_9_ and *f*_16_. ACLPSO-1 finds an unsatisfactory local optimum on *f*_14_ for all the runs, ACLPSO-2 cannot locate the global optimum or a near-optimum solution on *f*_3_, *f*_10_, *f*_11_, and *f*_14_ for all or some of the runs, and ACLPSO-3 is not able to effectively solve *f*_4_, *f*_6_, *f*_12_, *f*_13_, and *f*_14_ as the solutions found are unsatisfactory for all or some of the runs. The comparisons among ACLPSO, ACLPSO-1, ACLPSO-2, and ACLPSO-3 validate that the repairing of a particle's dimensional position when trespassing the dimensional search space, the dimensional independent and adaptive inertia weights and acceleration coefficients, and the dimensional independent and adaptive learning probabilities is appropriate to be employed by ACLPSO.

It can be seen from Table 4 that ACLPSO, in general, outperforms the literature PSO variants including CLPSO, ECLPSO, OLPSO-L, AGPSO, FLGPSO-QIW, and ALC-GPSO in terms of the mean global best fitness value results. CLPSO and ECLPSO both fail to obtain the global optimum or a near-optimum solution on *f*_3_, *f*_4_, *f*_12_, *f*_13_, and *f*_14_ for all the runs. ECLPSO fails additionally on *f*_10_ with the finding of an unsatisfactory local optimum for all the runs. As the mean and SD global best fitness value results of OLPSO-L, AGPSO, FLGPSO-QIW, and ALC-GPSO are directly copied from [7, 24], the symbol “—” represents an unavailable result in Table 4. We can see unsatisfactory mean global best fitness value results for OLPSO-L on *f*_10_, *f*_13_, and *f*_14_, for AGPSO on *f*_3_, *f*_4_, *f*_5_, *f*_12_, *f*_13_, *f*_15_, and *f*_16_, for FLGPSO-QIW on *f*_3_, *f*_4_, *f*_5_, *f*_6_, *f*_12_, *f*_13_, *f*_15_, and *f*_16_, and for ALC-GPSO on *f*_3_, *f*_9_, and *f*_10_. The accuracies of ACLPSO's mean global best fitness value results are the best on *f*_4_, *f*_5_, *f*_6_, *f*_7_, *f*_8_, *f*_9_, *f*_10_, *f*_11_, *f*_12_, *f*_13_, *f*_14_, and *f*_15_. The symbol “—” also appears in Table 5 and denotes a division by zero error. The *t*-test results are less than 0.05 on *f*_2_, *f*_3_, *f*_4_, *f*_10_, *f*_12_, *f*_13_, and *f*_14_; therefore, the global best fitness value results of ACLPSO are significantly different from those of ECLPSO on these 7 functions according to the statistics perspective. Based on the observations, though ECLPSO enhances the exploitation and convergence performance of CLPSO, the exploration performance of ECLPSO is as weak as that of CLPSO on some complex problems; and ACLPSO successfully addresses significantly bettering the exploration performance of ECLPSO, and ACLPSO is still good at exploitation and convergence, owing to the adoption of the dimensional independent and adaptive maximum velocities, inertia weights, acceleration coefficients, and learning probabilities.

ACLPSO and ECLPSO take advantage of more strategies than CLPSO to significantly achieve better performance; as a result, the mean execution time results of ACLPSO and ECLPSO are more than those of CLPSO on all the functions in Table 6. The differences are most noticeable on *f*_1_, *f*_2_, *f*_7_, *f*_8_, *f*_11_, *f*_15_, and *f*_16_, and the mean execution time of ACLPSO and that of ECLPSO are both about 300 to 500 ms more than that of CLPSO on each of these 7 functions. It must be pointed out that, for many real-world complex problems, the function evaluation or, in other words, evaluating the fitness of a position could be much time consuming, and accordingly, the execution time difference caused by more strategies of ACLPSO and ECLPSO than CLPSO would become relatively very small. The mean execution time results of ACLPSO are slightly more than those of ECLPSO on *f*_1_, *f*_2_, *f*_3_, *f*_7_, *f*_8_, *f*_11_, and *f*_13_, considerably more on *f*_4_, *f*_12_, *f*_14_, *f*_15_, and *f*_16_, slightly less on *f*_5_ and *f*_6_, and considerably less on *f*_9_ and *f*_10_, meaning that the dimensional independent and adaptive maximum velocities, inertia weights, acceleration coefficients, and leaning probabilities essentially do not increase execution time. With respect to ACLPSO, ECLPSO, and CLPSO, the mean execution time spent on a rotated function is more than that consumed on the corresponding original function, as can be observed from the pair of the mean execution time results on *f*_13_ and *f*_5_, the pair of *f*_14_ and *f*_9_, the pair of *f*_15_ and *f*_8_, and the pair of *f*_16_ and *f*_7_. The SD execution time results of ACLPSO, ECLPSO, and CLPSO are rather small as compared to the mean execution time results on all the functions.

The number of particles *N* is also a key parameter for ACLPSO. As can be seen from Table 7, setting *N* as 20 on *f*_4_ and *f*_15_ renders much worse mean and SD global best fitness value results, and ACLPSO cannot find the global optimum or a near-optimum solution for all or most of the runs because less particles lead to insufficient diversity. We can also observe from Table 7 that letting the learning probability-based coefficient *v* to be more than 0.05 on *f*_3_, the normative interval scaling coefficient *s* to be more than 0.1 on *f*_4_, *v* to be less than 0.3 on *f*_12_, and *s* to be less than 1.1 on *f*_14_ causes ACLPSO to be unable to find the optimum or a near-optimum solution for all or some of the runs. The mean and SD global best fitness value and execution time results of CLPSO on *f*_1_ and *f*_2_ in Table 8 indicate that simply by increasing the number of FEs to 500,000 or even increasing *N* to 80, CLPSO still cannot achieve high-accuracy mean global best fitness value as ECLPSO, while the mean execution time results of CLPSO are close to those of ECLPSO. The PbE and ALP strategies as well as the empirical values chosen for *N* and the best (*s*, *v*) combination are thus appropriate.

As shown in Figure 2, ECLPSO is liable to get stuck in premature stagnancy on *f*_3_, *f*_4_, *f*_12_, *f*_13_, and *f*_14_. The exploitation performance of ACLPSO is quite better than that of ECLPSO on *f*_2_. ACLPSO escapes from an unsatisfactory local optimum at the early stage of the search process on *f*_4_ and *f*_14_, at the middle stage on *f*_3_ and *f*_12_, and at the late stage on *f*_13_. According to Tables 2 and 3, ACLPSO takes the same best *s* values on *f*_3_, *f*_4_, and *f*_12_, the same best *v* values on *f*_4_, *f*_12_, *f*_13_, and *f*_14_, and the same best *s* values on *f*_13_ and *f*_14_; the mean number of exploitation valid dimensions' results is 30 for the best (*s*, *v*) combinations of ACLPSO on *f*_4_ and *f*_12_, slightly smaller than 30 on *f*_13_ and *f*_14_, and considerably smaller than 30 on *f*_3_. It is challenging to develop a unified setting for *s* and *v* based on the generation counter and the dimensional normative intervals.

## 6. Conclusions

In this paper, we have proposed ACLPSO for the purpose of further significantly mending the exploration performance of ECLPSO. ACLPSO introduces an independent inertia weight and an independent acceleration coefficient corresponding to each dimension and an independent learning probability for each particle on each dimension. ACLPSO determines the normative interval with respect to each dimension in each generation. Based on the dimensional normative intervals, ACLPSO adaptively adjusts the dimensional independent maximum velocities, inertia weights, acceleration coefficients, and learning probabilities. Experiments on a variety of unimodal, multimodal, shifted, rotated, and shifted rotated benchmark functions have demonstrated that ACLPSO successfully addresses exploration as well as exploitation and convergence as ACLPSO is able to derive the global optimum or a near-optimum solution on all the functions for all the runs with the normative interval scaling coefficient and the learning probability-based coefficient appropriately set. ACLPSO is a promising metaheuristic for global optimization. In the future, we plan to dig out more critical information inherently embodied in the search experience of the particles and try to develop a high-performance PSO variant based on ACLPSO with a unified parameter setting that works well on most global optimization problems and complex real-world applications, e.g., optimal operation of power systems [50, 51].

## Figures and Tables

**Table 1:** Benchmark functions.

Category	Benchmark function		*X* ^*∗*^	*f*(*X*^*∗*^)	Search space	Initialization space
	Name	Expression				
Unimodal	Sphere	*f*_1_(*X*)=∑_*d*=1_^*D*^*X*_*D*_^2^	{0}^*D*^	0	[−100,100]^*D*^	[−100,50]^*D*^
	Schwefel P2.22	*f*_2_(*X*)=∑_*d*=1_^*D*^|*X*_*d*_|+∏_*d*=1_^*D*^|*X*_*d*_|	{0}^*D*^	0	[−10,10]^*D*^	[−10,5]^*D*^
	Rosenbrock	*f*_3_(*X*)=∑_*d*=1_^*D*−1^(100(*X*_*d*+1_ − *X*_*D*_^2^)^2^+(*X*_*d*_ − 1)^2^)	{1}^*D*^	0	[−10,10]^*D*^	[−10,10]^*D*^
	Schwefel P1.2	*f*_4_(*X*)=∑_*d*=1_^*D*^(∑_*j*=1_^*d*^*X*_*j*_)^2^	{0}^*D*^	0	[−100,100]^*D*^	[−100,50]^*D*^

Multimodal	Rastrigin	*f*_5_(*X*)=10 *D*+∑_*d*=1_^*D*^(*X*_*D*_^2^ − 10 cos(2*πX*_*d*_))	{0}^*D*^	0	[−5.12, 5.12]^*D*^	[−5.12, 2]^*D*^
	Noncontinuous Rastrigin	$\begin{array}{c} f_{6}\left(X\right)=f_{5}\left(Y\right),\\ Y_{d}=\left\{\begin{array}{c} X_{d},\text{ if }\left|X_{d}\right|<0.5\\ \text{Round}\left(2X_{d}\right)/2\text{, otherwise} \end{array}\right) \end{array}$	{0}^*D*^	0	[−5.12, 5.12]^*D*^	[−5.12, 2]^*D*^
	Ackley	$\begin{array}{c} f_{7}\left(X\right)=-20\;\exp\left(-0.2\sqrt{\left(1/D\right)\sum_{d=1}^{D}X_{D}^{2}}\right)-\\ \exp\left(\left(1/D\right)\sum_{d=1}^{D}\cos\left(2\pi X_{d}\right)\right)+20+e \end{array}$	{0}^*D*^	0	[−32,32]^*D*^	[−32,20]^*D*^
	Griewank	$\begin{array}{c} f_{8}\left(X\right)=1/4000\sum_{d=1}^{D}X_{D}^{2}-\prod_{d=1}^{D}\cos\left(X_{d}|/|\sqrt{d}\right)+1 \end{array}$	{0}^*D*^	0	[−600,600]^*D*^	[−600,200]^*D*^
	Schwefel	$f_{9}\left(X\right)=418.9829D-\sum_{d=1}^{D}X_{d}\sin\left(\sqrt{\left|X_{d}\right|}\right)$	{420.96}^*D*^	0	[−500,500]^*D*^	[−500,500]^*D*^

Shifted	Shifted Rosenbrock	*f*_10_(*X*)=*f*_3_(*Y*)+390, *Y*=*X* − *Z*+1	*Z*	390	[−100,100]^*D*^	[−100,100]^*D*^
	Shifted Griewank	*f*_11_(*X*)=*f*_8_(*Y*), *Y*=*X* − *Z*	*Z*	0	[−600,600]^*D*^	[−600,600]^*D*^
	Shifted Schwefel P1.2	*f*_12_(*X*)=*f*_4_(*Y*) − 450, *Y*=*X* − *Z*	*Z*	−450	[−100,100]^*D*^	[−100,100]^*D*^

Rotated	Rotated Rastrigin	*f*_13_(*X*)=*f*_5_(*Y*), *Y*=*XO*	{0}^*D*^	0	[−5.12, 5.12]^*D*^	[−5.12, 2]^*D*^
	Rotated Schwefel	$\begin{array}{c} f_{14}\left(X\right)=f_{9}\left(Y\right),\\ Y_{d}=\left\{\begin{array}{c} Z_{d}\sin\left(\sqrt{\left|Z_{d}\right|}\right),\text{ if }\left|Z_{d}\right|\leq 500\\ 0,\text{ otherwise} \end{array}\right),\\ Z=\left(X-{\left\{420.96\right\}}_{1\times D}\right)O+{\left\{420.96\right\}}_{1\times D} \end{array}$	{420.96}^*D*^	0	[−500,500]^*D*^	[−500,500]^*D*^
	Rotated Griewank	*f*_15_(*X*)=*f*_8_(*Y*), *Y*=*XO*	{0}^*D*^	0	[−600,600]^*D*^	[−600,200]^*D*^

Shifted rotated	Shifted rotated Ackley	*f*_16_(*X*)=*f*_7_(*Y*), *Y*=(*X* − *Z*)*O*	*Z*	0	[−32,32]^*D*^	[−32,32]^*D*^

**Table 2:** Mean and standard deviation global best fitness value results of ACLPSO with different values for the normative interval scaling coefficient *s* and the learning probability-based coefficient *v* on all the benchmark functions.

Function	Global best fitness value	ACLPSO			
		I: *s* = 1.1; *v* = 0.05	II: *s* = 1.1; *v* = 0.3	III: *s* = 0.1; *v* = 0.05	IV: *s* = 0.1; *v* = 0.3
*f*_1_	Mean	1.23*E* − 10	3.37*E* − 105	2.87*E* − 12	**3.28E** **−** **115**
	SD	6.08*E* − 10	9.36*E* − 105	8.18*E* − 12	**6.11E** **−** **115**

*f*_2_	Mean	1.48*E* − 17	4.74*E* − 22	3.09*E* − 29	**7.25E** **−** **37**
	SD	2.19*E* − 17	9.70*E* − 22	4.27*E* − 29	**6.84E** **−** **37**

*f*_3_	Mean	4.23	6.56*E*1	**1.84**	2.61*E*1
	SD	2.43	2.49*E*1	**1.63**	2.87*E*1

*f*_4_	Mean	1.56*E*4	1.71*E*4	3.39*E*2	**7.87E** **−** **2**
	SD	3.44*E*3	8.24*E*3	8.84*E*1	**8.72E** **−** **2**

*f*_5_	Mean	**0**	1.55	5.17*E* − 1	3.02
	SD	**0**	9.12*E* − 1	5.83*E* − 1	1.45

*f*_6_	Mean	4.19*E* − 1	**0**	5.66*E* − 1	2.00*E* − 1
	SD	7.19*E* − 1	**0**	6.63*E* − 1	4.08*E* − 1

*f*_7_	Mean	1.92*E* − 6	**3.11E** **−** **15**	1.15*E* − 8	3.25*E* − 15
	SD	6.96*E* − 6	**0**	5.77*E* − 8	7.11*E* − 16

*f*_8_	Mean	3.35*E* − 8	**0**	1.87*E* − 8	1.38*E* − 11
	SD	3.88*E* − 8	**0**	3.45*E* − 8	4.56*E* − 11

*f*_9_	Mean	**3.82E** **−** **4**	4.29*E*2	2.23*E*2	5.87*E*2
	SD	**9.15E** **−** **7**	2.71*E*2	1.38*E*2	2.04*E*2

*f*_10_	Mean	**4.05E2**	5.12*E*2	4.23*E*2	5.86*E*2
	SD	**1.60E1**	1.02*E*2	2.32*E*1	2.56*E*2

*f*_11_	Mean	1.26*E* − 7	**0**	1.28*E* − 7	1.96*E* − 3
	SD	3.30*E* − 7	**0**	2.34*E* − 7	8.01*E* − 3

*f*_12_	Mean	4.19*E*3	3.26*E*2	6.37*E*2	**−4.48E2**
	SD	9.97*E*2	2.28*E*2	2.59*E*2	**3.04**

*f*_13_	Mean	3.65*E*1	**3.25**	2.04*E*1	1.39*E*1
	SD	7.40	**1.88**	3.70	2.53

*f*_14_	Mean	5.78*E*2	**2.53E2**	1.35*E*3	1.32*E*3
	SD	4.00*E*2	**2.09E2**	1.85*E*2	2.19*E*2

*f*_15_	Mean	2.05*E* − 3	**5.33E** **−** **17**	1.65*E* − 3	2.76*E* − 3
	SD	2.71*E* − 3	**6.51E** **−** **17**	2.70*E* − 3	5.91*E* − 3

*f*_16_	Mean	2.73*E* − 5	3.96*E* − 15	1.74*E* − 6	**3.82E** **−** **15**
	SD	9.24*E* − 5	1.55*E* − 15	6.94*E* − 6	**1.45E** **−** **15**

**Table 3:** Mean and standard deviation number of exploitation valid dimensions' results of ACLPSO with different (*s*, *v*) combinations on all the benchmark functions.

Function	Number of exploitation valid dimensions	ACLPSO			
		I: *s* = 1.1; *v* = 0.05	II: *s* = 1.1; *v* = 0.3	III: *s* = 0.1; *v* = 0.05	IV: *s* = 0.1; *v* = 0.3
*f*_1_	Mean	26.4	30	24	30
	SD	9.95	0	12.25	0

*f*_2_	Mean	30	30	30	30
	SD	0	0	0	0

*f*_3_	Mean	1.08	29.4	6.04	30
	SD	5.40	1.83	10.13	0

*f*_4_	Mean	0	0	0	30
	SD	0	0	0	0

*f*_5_	Mean	30	30	10.48	29.72
	SD	0	0	3.08	0.46

*f*_6_	Mean	0	0.2	0	0.12
	SD	0	0.41	0	0.44

*f*_7_	Mean	27.56	30	28.8	30
	SD	8.30	0	6.00	0

*f*_8_	Mean	2.2	30	2.36	29.52
	SD	6.09	0	5.16	1.16

*f*_9_	Mean	22.8	30	5.24	30
	SD	13.08	0	7.90	0

*f*_10_	Mean	6.36	29.76	7.4	29.96
	SD	11.55	0.72	12.12	0.2

*f*_11_	Mean	0.68	30	1.6	29.6
	SD	3.00	0	3.88	0.82

*f*_12_	Mean	0	6.28	0	30
	SD	0	6.28	0	0

*f*_13_	Mean	0	25.44	0	8.72
	SD	0	8.73	0	7.83

*f*_14_	Mean	3.28	28.36	0	27.12
	SD	5.12	3.62	0	7.37

*f*_15_	Mean	3.92	30	2.52	29.56
	SD	5.10	0	4.74	1.08

*f*_16_	Mean	26.4	30	27.6	30
	SD	9.95	0	8.31	0

**Table 4:** Mean and standard deviation global best fitness value results of ACLPSO with the best (*s*, *v*) combination and other PSO variants on all the benchmark functions.

Function	Global best fitness value	ACLPSO	ACLPSO-1	ACLPSO-2	ACLPSO-3	CLPSO	ECLPSO	OLPSO-L	AGPSO	FLGPSO-QIW	ALC-GPSO
*f*_1_	Mean	3.28*E* − 115	1.85*E* − 113	3.38*E* − 114	2.19*E* − 12	3.11*E* − 14	2.74*E* − 93	1.11*E* − 38	1.70*E* − 1	4.07*E* − 48	1.67*E* − 161
	SD	6.11*E* − 115	9.13*E* − 113	5.01*E* − 114	7.47*E* − 12	5.98*E* − 14	4.71*E* − 93	1.28*E* − 38	1.28*E* − 1	1.12*E* − 47	8.20*E* − 161

*f*_2_	Mean	7.25*E* − 37	1.14*E* − 36	6.25*E* − 36	1.51*E* − 32	8.22*E* − 10	4.24*E* − 30	7.67*E* − 22	—	—	1.16*E* − 90
	SD	6.84*E* − 37	2.06*E* − 36	6.52*E* − 36	1.82*E* − 32	3.84*E* − 10	3.02*E* − 30	5.63*E* − 22	—	—	4.14*E* − 90

*f*_3_	Mean	1.84	2.67	8.64	2.53	3.73*E*1	3.25*E*1	1.26	3.07*E*1	2.47*E*1	7.61
	SD	1.63	2.95	8.04	4.16	2.04*E*1	1.63*E*1	1.40	1.35*E*1	5.07*E* − 1	6.66

*f*_4_	Mean	7.87*E* − 2	4.41*E* − 2	1.07*E* − 1	3.63*E*2	5.42*E*2	5.46*E*2	—	3.87*E*2	5.27*E*1	—
	SD	8.72*E* − 2	4.80*E* − 2	1.43*E* − 1	9.89*E*1	1.81*E*2	1.60*E*2	—	1.39*E*2	2.66*E*1	—

*f*_5_	Mean	0	0	1.48*E* − 9	1.43*E* − 5	1.59*E* − 6	0	0	1.02	2.64	2.53*E* − 14
	SD	0	0	5.70*E* − 9	6.78*E* − 5	1.15*E* − 6	0	0	1.01	1.61	1.38*E* − 14

*f*_6_	Mean	0	0	0	5.80*E* − 1	0	1.12*E* − 3	—	6.15*E* − 2	3.70	1.25*E* − 11
	SD	0	0	0	6.78*E* − 1	0	4.27*E* − 3	—	1.82*E* − 1	2.14	6.75*E* − 11

*f*_7_	Mean	3.11*E* − 15	3.11*E* − 15	3.11*E* − 15	2.05*E* − 4	5.10*E* − 8	3.11*E* − 15	4.14*E* − 15	6.53*E* − 2	4.77*E* − 14	1.15*E* − 14
	SD	0	0	0	8.86*E* − 4	3.38*E* − 8	0	0	3.34*E* − 2	3.94*E* − 14	2.94*E* − 14

*f*_8_	Mean	0	0	0	4.60*E* − 9	1.58*E* − 9	0	0	1.90*E* − 1	1.97*E* − 3	1.22*E* − 2
	SD	0	0	0	8.73*E* − 9	2.60*E* − 9	0	0	1.90*E* − 1	1.97*E* − 3	1.58*E* − 2

*f*_9_	Mean	3.82*E* − 4	3.82*E* − 4	3.82*E* − 4	3.82*E* − 4	3.82*E* − 4	3.82*E* − 4	3.82*E* − 4	—	—	2.10*E*1
	SD	9.15*E* − 7	4.26*E* − 10	4.51*E* − 10	4.66*E* − 7	1.54*E* − 12	5.04*E* − 12	0	—	—	5.41*E*1

*f*_10_	Mean	4.05*E*2	4.06*E*2	5.05*E*2	4.25*E*2	4.13*E*2	5.22*E*2	2.60*E*3	—	—	2.18*E*1
	SD	1.60*E*1	9.45	6.17*E*1	4.09*E*1	2.26*E*1	3.07*E*1	2.40*E*1	—	—	4.04*E*1

*f*_11_	Mean	0	0	2.49*E* − 1	2.30*E* − 8	2.26*E* − 9	0	—	0	4.85*E* − 3	
	SD	0	0	4.68*E* − 1	5.02*E* − 8	4.30*E* − 9	0	—	0	4.12*E* − 3	—

*f*_12_	Mean	−4.48*E*2	−4.48*E*2	−4.49*E*2	6.07*E*2	1.22*E*3	1.19*E*3	—	3.33*E*2	1.56*E*2	7.34*E* − 10
	SD	3.04	1.95	1.26	2.02*E*2	3.63*E*2	3.70*E*2	—	1.73*E*2	7.97*E*1	2.73*E* − 12

*f*_13_	Mean	3.25	5.10	5.22	4.16*E*1	2.86*E*1	2.50*E*1	5.34*E*1	4.52*E*2	3.76*E*2	—
	SD	1.88	2.27	3.05	6.76	4.00	4.58	1.34*E*1	1.62*E*1	8.88	—

*f*_14_	Mean	2.53*E*2	1.27*E*3	3.56*E*2	8.83*E*2	1.29*E*3	1.18*E*3	3.13*E*3	—	—	—
	SD	2.09*E*2	2.09*E*2	4.89*E*2	4.17*E*2	1.38*E*2	1.17*E*2	1.24*E*3	—	—	—

*f*_15_	Mean	5.33*E* − 17	1.80*E* − 11	1.42*E* − 13	1.14*E* − 4	5.76*E* − 5	1.49*E* − 3	4.19*E* − 8	1.01*E*2	1.40	—
	SD	6.51*E* − 17	8.98*E* − 11	7.08*E* − 13	1.71*E* − 4	5.25*E* − 5	4.37*E* − 3	2.06*E* − 7	7.50*E*1	4.35*E* − 1	—

*f*_16_	Mean	3.82*E* − 15	7.45*E* − 2	3.96*E* − 15	3.68*E* − 15	1.03*E* − 6	3.96*E* − 15	—	2.06*E*1	2.10*E*1	—
	SD	1.45*E* − 15	2.58*E* − 1	1.55*E* − 15	1.33*E* − 15	1.54*E* − 6	1.55*E* − 15	—	1.24*E* − 1	5.25*E* − 2	—

**Table 5:** Two-tailed *t*-test results for the comparison of the global best fitness value results between ACLPSO with the best (*s*, *v*) combination and ECLPSO on all the benchmark functions.

Function	*f*_1_	*f*_2_	*f*_3_	*f*_4_	*f*_5_	*f*_6_	*f*_7_	*f*_8_
*t*-test	—	3.01*E* − 7	1.56*E* − 9	6.68*E* − 15	—	0.20	0.33	—

Function	*f*_9_	*f*_10_	*f*_11_	*f*_12_	*f*_13_	*f*_14_	*f*_15_	*f*_16_

*t*-test	0.06	1.21*E* − 18	—	1.77*E* − 17	8.49*E* − 21	3.01*E* − 21	0.10	0.74

**Table 6:** Mean and standard deviation execution time results of ACLPSO with the best (*s*, *v*) combination, ECLPSO, and CLPSO on all the benchmark functions.

Function			*f*_1_	*f*_2_	*f*_3_	*f*_4_	*f*_5_	*f*_6_	*f*_7_	*f*_8_
ACLPSO	Execution time (in ms)	Mean	784.20	847.88	485.52	765.48	617.16	545.16	823.52	888.64
		SD	32.11	37.07	63.98	50.57	26.41	39.47	41.14	39.62

ECLPSO	Execution time (in ms)	Mean	732.20	794.84	474.76	550.76	680.96	563.28	795.84	827.12
		SD	24.49	28.65	22.78	26.11	31.31	26.37	30.06	40.16

CLPSO	Execution time (in ms)	Mean	322.68	363.08	370.40	411.36	448.40	428.16	443.64	497.96
		SD	16.75	22.64	22.28	20.09	21.93	21.40	22.16	15.45

Function			*f*_9_	*f*_10_	*f*_11_	*f*_12_	*f*_13_	*f*_14_	*f*_15_	*f*_16_
ACLPSO	Execution time (in ms)	Mean	672.04	531.96	887.88	745.08	821.24	1138.20	1102.04	1095.20
		SD	46.41	86.05	40.16	39.98	42.10	79.25	37.37	46.11

ECLPSO	Execution time (in ms)	Mean	853.60	728.88	848.08	576.72	804.80	945.84	1010.16	1002.08
		SD	58.39	36.64	90.89	30.85	38.12	33.49	60.72	39.65

CLPSO	Execution time (in ms)	Mean	487.24	380.84	519.04	435.84	660.28	794.28	738.80	663.08
		SD	15.41	26.48	23.56	33.77	40.25	34.91	20.50	40.10

**Table 7:** Mean and standard deviation global best fitness value results of ACLPSO with other parameter settings on *f*_3_, *f*_4_, *f*_12_, and *f*_14_.

Function	Parameter setting	Global best fitness value	
		Mean	SD
*f*_3_	*N* = 40, *s* = 0.1, *v* = 0.2	1.89*E*1	2.66*E*1
	*N* = 40, *s* = 0.1, *v* = 0.1	1.44*E*1	2.24*E*1

*f*_4_	*N* = 20, *s* = 0.1, *v* = 0.3	6.32*E*2	3.62*E*2
	*N* = 40, *s* = 0.3, *v* = 0.3	1.72*E*2	1.34*E*2
	*N* = 40, *s* = 0.9, *v* = 0.3	2.69*E*4	5.48*E*3

*f*_12_	*N* = 40, *s* = 0.1, *v* = 0.1	2.15*E*2	1.90*E*2
	*N* = 40, *s* = 0.1, *v* = 0.2	−4.18*E*2	1.18*E*2

*f*_14_	*N* = 20, *s* = 1.1, *v* = 0.3	7.56*E*2	5.21*E*2
	*N* = 40, *s* = 0.9, *v* = 0.3	4.25*E*2	4.34*E*2
	*N* = 40, *s* = 0.3, *v* = 0.3	5.61*E*2	6.43*E*2

**Table 8:** Mean and standard deviation global best fitness value and execution time results of CLPSO with other parameter settings on *f*_1_ and *f*_2_.

Function	Parameter setting	Global best fitness value		Execution time (in ms)	
		Mean	SD	Mean	SD
*f*_1_	*N* = 40; the number of FEs is 500,000	7.13*E* − 29	2.41*E* − 28	815.32	27.00
	*N* = 80; the number of FEs is 500,000	3.13*E* − 19	5.15*E* − 19	805.48	28.88

*f*_2_	*N* = 40; the number of FEs is 500,000	9.39*E* − 22	1.72*E* − 21	844.08	13.77
	*N* = 80; the number of FEs is 500,000	8.38*E* − 13	2.69*E* − 13	861.88	22.36

## Data Availability

The data used to support the findings of this study are available from the corresponding author upon request.
